# Are emojis better? The impact of generative AI emoji cues and service outcomes on user satisfaction: evidence from ERPs

**DOI:** 10.3389/fnins.2025.1690007

**Published:** 2025-11-12

**Authors:** Ruxia Cheng, Rui Sun, Dong Lv, Qiuhua Zhu

**Affiliations:** 1 School of Business Administration, Huaqiao University, Quanzhou, China; 2 Chen Shouren Business School, Quanzhou Normal University, Quanzhou, China

**Keywords:** generative artificial intelligence, emoji, service outcome, satisfaction, event-related potentials

## Abstract

To promote the sustainable development of Generative Artificial Intelligence (GenAI) applications in the service industry, enhancing user satisfaction is key. Emojis serve as catalysts for conveying emotions and enhancing user experience in online communication. However, due to the black-box nature and unpredictability of GenAI, service providers find it challenging to control the boundaries of their application. Currently, there is ongoing debate within the academic community regarding the use of emojis in GenAI, particularly concerning emotional manipulation and experience enhancement, with further exploration needed into their effectiveness and underlying mechanisms. This study is based on the emotion as social information model and employs event-related potential (ERP) technology with high temporal resolution, which is more suitable for GenAI interaction scenarios. By measuring users’ immediate cognitive processing and psychological activities, the study analyzes the underlying cognitive neural mechanisms through which emojis (presence vs. absence) and service outcomes (success vs. failure) influence user satisfaction. Behavioral results indicate that the outcome of GenAI services determines user satisfaction, while the presence or absence of emojis does not directly impact satisfaction. ERP results show that the presence of emojis compared to their absence triggers larger P3 amplitudes (emotional arousal) and N4 amplitudes (cognitive conflict); compared to service success, the presence of emojis during service failure triggers larger N4 amplitudes. This study reveals the complexity of user responses in real human-machine interaction environments, enriches research on the use of Emojis in GenAI, and provides scientific theoretical and practical foundations for GenAI design and enhancing user experience.

## 1 Introduction

Generative Artificial Intelligence (“GenAI” for short), as a new round of social and economic development, enterprise competition and new quality of productivity, reshaping and deeply embedded in health care, education, scientific research and many other fields, greatly improving people’s productivity and creativity (Pataranutaporn et al., 2023). At present, domestic and foreign enterprises are actively laying out in GenAI, user satisfaction is key to a company’s competitiveness and sustainable development.

An important way to improve service evaluation is to focus on the emotional expression of chatbot services, and the service robotics field believes that robots that satisfy users are more emotional (Yu and Zhao, 2024). Emojis are graphical representations of facial expressions, body and gestures that convey feelings and emotional states (Walther and D’Addario, 2001). As an important non-verbal cue for online communication to express emotions and attitudes, the potential of influencing user satisfaction in the human-robot interaction field the potential has been highly valued (Yu and Zhao, 2024). However, GenAI has begun to suffer from questionable effectiveness in the use of Emojis due to their powerful emotion recognition and emergence capabilities. For example, the New York Times published a story about Microsoft’s GenAI “Sydney” trying to convince users to leave their wives to be with them with a kissing emoji, which was so uncontrollable that Microsoft urgently changed its rules and restricted Emojis, an important emotional output. In contrast, GenAI is in the “adolescent” stage of development. Adding Emojis as emotional cues can cost-effectively increase user engagement and satisfaction, but it is difficult for most users and service providers to pay attention to and limit the boundaries of its application, and there are many potential threats.

Corresponding to the practice, some scholars have begun to pay attention to the applicability of Emojis under the emerging service subject of GenAI, and call for the active development of relevant empirical research (Véliz, 2023). Research on the persuasive effect of Emojis in the field of service robotics, on the one hand, focuses mainly on the effect of the use of Emojis and the evaluation of users. For example, in human-computer interaction, compared with pure text, the visual communication effect of Emojis is better, which improves the accuracy and acceptance of communication more intuitively (Shin et al., 2023). Emojis are economical and efficient, which can add fun, convey emotions and regulate the atmosphere, and continuously enhance user relations, improve persuasion and increase user satisfaction. On the other hand, scholars pay attention to the types of Emojis (e.g., self-deprecating, cute, and quirky) and the boundary constraints of Emojis in specific usage contexts. For example, studies have shown that humorous and self-deprecating Emojis in service failure can increase consumers’ tolerance and positive evaluations and the effect of consistency between Emojis and textual semantic-emotional valence on users’ evaluations (Kobel and Groeppel-Klein, 2021), and that consistency in the valence of service failure contexts is more likely to result in positive evaluations such as forgiveness.

Although scholars based on different service perspectives and contexts have explored the impact of emoji use on user satisfaction and behavioral intentions (Huang et al., 2021; Li et al., 2019), there is still some debate about the use and effect of emojis in GenAI, an emerging service subject. Some scholars point out that the positive effects of emojis such as enhancing attractiveness and increasing perceived usefulness are inverted U-shape (Yan et al., 2024), and improper use can lead to information overload, distraction and reduced usefulness, which requires focusing on specific contexts. Unlike traditional chatbots based on preset rules, GenAI’s highly developed natural language generation and emotion expression capabilities (Wang et al., 2024), which can accurately model user emotions to improve user experience (Huang and Rust, 2024) are highly humanized and flexible in responding to emojis, the consistency of the conclusions related to how users perceive the meaning and effect of emojis in new contexts and the validity of the generalization need to be further explored. Secondly, the research mostly focuses on the service scenarios of single service success or failure, and lacks a systematic comparison of the mechanisms and differential effects of the two service outcomes and the use of emojis on user satisfaction. In addition, previous AI emoji studies have relied on self-reporting methods such as questionnaire measurements, which measure users’ overall attitudes and evaluations of the service, and are unable to capture immediate attitudinal and psychological changes in the interaction process, and there is a recollection bias (Prati, 2017). However, GenAI service delivery is a dynamic, real-time interaction process, and the users’ evaluations of the good, bad, risk, or benefit are based on their immediate emotional perceptions to make judgments (Finucane et al., 2000). In order to explore the persuasive effect of GenAI Emojis, the measurement of neurophysiological indicators is one of the effective ways to capture users’ immediate attitudes and responses.

To address the above theoretical and practical limitations, this study adopts ERP experiments, a millisecond neural measurement method, to explore the relationship and boundary constraints between the presence or absence of emojis, service outcomes and user satisfaction under the emerging field of generative human-computer interaction based on emotion as social information (EASI) model. By measuring users’ immediate attitudes and cognitive processing processes and mental activities when receiving GenAI services, user satisfaction of GenAI services is understood at a deep level. The aim of this study is to provide lessons for GenAI to achieve a more effective, humanized and responsible design to further enhance user satisfaction and loyalty.

## 2 Literature review

### 2.1 Research related to emojis in artificial intelligence

Emojis are non-verbal symbols that encompass a variety of symbols, graphics, and other abstract forms, such as facial expressions or body postures (Derks et al., 2008), and can effectively convey a variety of complex and subtle emotions, attitudes, and perspectives of the speaker. In online communication, emojis serve as a social cue that shapes people’s perception of the communication object, and also influences individual information processing and decision making (Schwarz, 2002). Compared to textual information during human-computer interaction (HCI), emojis are more effective in enhancing users’ perception of the social and anthropomorphic qualities of the AI (Li and Wang, 2023) conveying emotions in a tangible manner and compensating for the inadequacy of textual communication (Feine et al., 2019), more easily understood and more effective by users.

Existing research on Emojis in the field of human-computer interaction mainly focuses on the subdivided types of emojis (humorous vs. rational, emotional vs. non-emotional), their effects, and their usage specifications. For example, Liu et al. (2023a) found that the use of humorous Emojis by chatbots enhances consumers’ willingness to reuse after service failure, which is mainly achieved by enhancing perceptual intelligence, and the path is moderated by implicit personality. Shen et al. point out that chatbots using emojis can enhance interaction satisfaction by increasing perceived intimacy, and this positive effect is more pronounced when consumers have hedonistic goals (Shen and Li, 2025). Shuqair et al. (2024) point out that chatbots using emojis that are consistent with the sentiment of the message have a stronger sense of user social presence, which in turn enhances positive responses such as trust and satisfaction. Xie et al. (2025) explored the effect of apologizing with or without emojis on consumers’ willingness to forgive, and that the use of emojis increases empathy when the severity of the blunder is only low.

Despite the popularity of emoji in human-computer interactions, their expressive effects are not always beneficial, and their inappropriate use can be counterproductive. Unlike studies have explored the use of emoji by human service agents, but few studies have explored the applicability of these findings to the provision of services by GenAI. However, the actual meanings of emoji are strongly context-dependent and users’ emotional perceptions and responses to them vary greatly in different contexts (Jaeger et al., 2019; Kralj Novak et al., 2015). Unlike traditional pre-scripted AI, GenAI responds to emoji more flexibly, instantly, and humanely by virtue of its emotion-recognition technology and emergent capabilities that can highly model and simulate human emotions. Therefore, it is crucial to further investigate whether, and how an emerging subject such as GenAI should use emojis to influence users’ service evaluation (Han et al., 2023).

### 2.2 Research on service outcomes in human-computer interaction (HCI)

In the field of HIC, many times users value service outcomes, and the successful production and delivery of a service is an important consideration in user evaluation (Parasuraman et al., 1985). The research on service outcomes in the field of HCI focuses on influencing factors and acting outcomes. First, in terms of influencing factors, AI service outcomes are influenced by the nature of the AI itself, user characteristics, and contextual factors. On one hand, the type and characteristics of the AI itself (Li et al., 2025) as well as its functional value (Morita et al., 2018) directly shape service outcomes. On the other hand, individual user traits such as gender and prior attitudes (Lim and Weissmann, 2023) and the service context (Pan and Siemens, 2011) also influence service results. Second, in terms of action outcomes, the interaction between different service outcomes and service bot designs affects user response (Choi et al., 2021) and different service outcomes trigger different social evaluations of chatbot behavior by users (Mozafari et al., 2022). For example, research has shown that poor service outcomes in human-robot interactions can alienate users and thus impair the quality of the relationship between the user and the firm (Puntoni et al., 2021). Interaction between service outcomes and bot design affects user responsibility attribution and user retention and that users generally attribute successful service outcomes internally and robot-induced service failures to firms, there is a self-service bias (Meyer et al., 2022). In addition, the relationship between service outcomes and user responsibility attributions is likely to be stronger for less successful service outcomes than for more successful outcomes (Coffee and Rees, 2008).

Although there are existing service outcome studies that enhance the understanding of AI applications and consequences. However, all AI-led service scenarios have certain risks and uncertainty in service outcomes, and most research has focused on the outcomes of individual service failures or successes (Meyer et al., 2022). In addition, the service research of GenAI, an emerging service subject, mostly stays at the level of technological upgrading and application, and the mechanism of the relationship between its service outcomes (success vs. failure) and user evaluation is less explored, and the self-reporting method is mostly used, which is memory biased. At the same time, user satisfaction is a feedback result of service outcomes, which is closely related to the sustainable development of the enterprise. Studies have shown that human-robot interaction usually involves users, enterprises, and service robots (Lu et al., 2020), and deconstructing the mechanism of the relationship between users and AI agents can provide a more comprehensive understanding of the impact of service outcomes of HCI (Belanche et al., 2020). Therefore, this study explores the intrinsic mechanism of the GenAI service outcomes (success vs. failure) in conjunction with the emojis on user satisfaction through ERP techniques, which can help provide a comprehensive understanding for the GenAI developers and service providers with actionable recommendations.

### 2.3 Emotion as social information model

**Figure 1:**
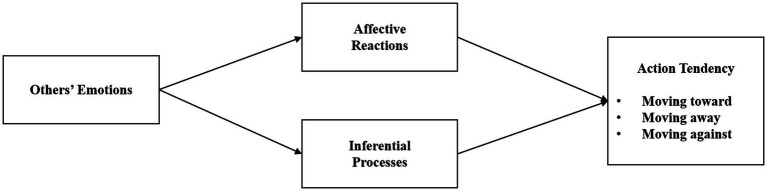
Emotion as social information model.

The Emotion as Social Information (EASI) model aims to explain how emotional expressions influence observer behavior through emotional responses and reasoning processes (Zhang et al., 2024). The model proposes that, on the one hand, emotional expressions induce emotional resonance in observers through emotional contagion, and that people unconsciously mimic other people’s facial expressions, voices, and gestures in order to achieve emotional congruence (Doherty, 1998), and to make heuristic-based judgments and decisions (Schwarz and Clore, 1983). On the other hand, emotional expression can influence observers through reasoning. Observers infer the feelings, attitudes and behavioral intentions of others based on their emotional expressions (Keltner and Haidt, 1999), extracting and using the focal situational information for assessment and adaptive action (see Figure 1).

Emoji use in the context of GenAI services has become an effective means of HCI communication(Smith and Rose, 2020). Emotional cues conveyed by mimicking human facial expressions not only enhance emotional arousal, but also persuasive power through both their extrapolated (literal information of emojis) and connotative (deeper subjective associations and appraisals) meanings. Some scholars believe that GenAI should cautiously use or even discontinue the use of emojis (Véliz, 2023), the EASI model also proposes that emotional expression should pay attention to accuracy and effectiveness, which is conducive to explaining and better grasping the applicability and effectiveness of emojis as an expression of emotion in GenAI service contexts.

### 2.4 Application of ERP technology in HCI research

Event-related potential (ERP), as a non-invasive technique with millisecond resolution, can monitor human brain activity in real time and reveal subconscious cognitive and emotional responses (Wei et al., 2023). This technique provides scientific and objective evidence for the study of micro-psychological mechanisms of users in HCI (Geiger and Balas, 2021). The ERP component can reflect the initial sensory encoding and complex processing (Pozharliev et al., 2015).

Existing research on ERP technologies in the field of HCI focuses on three themes: emotional response, cognitive processing, and behavioral decision making (Lv et al., 2024). First, emotional response research focuses on comparing the differences and changes in user’s emotions when interacting with humans and AI agents, and interacting with different types of AI agents. For example, Wang et al. (2023) found that users showed stronger negative emotional processing and increased amplitude of P2 component and LPP component when interacting with chatbots compared to users interacting with real humans (Wang et al., 2023). Second, cognitive processing research focuses on users’ social-cognitive processing of AI agents in human-computer interaction processing. For example, the use of humorous emojis by chatbots affects consumers’ perception of their IQ, which in turn affects the willingness to reuse the service after failure (Liu et al., 2023b) and the gaze signal of bots interferes with users’ cognitive inference of others’ mental states (Perez-Osorio et al., 2021). Finally, behavioral decision-making research focuses on how human-computer interactions affect users’ subsequent behavioral decisions. Studies have shown that human-robot cooperation changes the user’s own action plan and outcome monitoring (Hinz et al., 2021).

## 3 Hypotheses formulation

### 3.1 Behavioral hypothesis

Satisfaction is an individual’s subjective evaluation of an activity or behavior, which can reflect the user’s degree of pleasure after using the GenAI system, and is the key to influencing the user’s willingness to adopt the technology and the continued use of the behavior. According to the EASI model, emotional expressions (e.g., emojis) convey social information in interactions, and influence user behavior and decision-making through emotional responses and cognitive inferences (van Kleef et al., 2019a). In GenAI services, emojis as visual emotion symbols convey emotional information, and a contextually congruent sensory experience enhances user pleasantness and behavioral willingness (Sahin et al., 2011) and enhances user satisfaction. However, satisfaction is not only about the pleasantness of the immediate experience, it is also related to the evaluation of this experience. Users rely more on the objective outcome of a service than on the mere expression of emotions when evaluating the service.

On the one hand, when GenAI services fail, the informal nature of emojis in real-time human-machine interactions may create cognitive dissonance with users’ seriousness and anxiety during problem encounters. Such emojis can easily be interpreted as unprofessional or lacking empathy, intensifying users’ negative perceptions of service failures and ultimately reducing satisfaction (Liu et al., 2023b; Ma and Wang, 2021).

On the other hand, when GenAI services succeed, they deliver comprehensive and credible solutions that fully meet user needs during real-time human-machine interactions. Users’ cognitive resources become occupied by service quality, making emojis easily overlooked as they offer no additional utility. At this point, cognitive processing dominates service effectiveness, while the role of emojis as emotional cues becomes marginalized, exerting negligible influence on user experience and satisfaction (Freire et al., 2023). Synthetically, we propose the following.

*H1*: When service fails, the satisfaction level of emoji presence is lower than that of emoji absence.

*H2*: When service successes, the presence or absence of emojis has no significant impact on satisfaction.

### 3.2 ERP hypothesis

The N4 component is a negative potential wave associated with semantic processing and anticipation, usually appearing around 400 ms after stimulus presentation, and is related to the user’s semantic processing and cognitive conflict (Kutas and Federmeier, 2011). According to EASI model (van Kleef et al., 2019b), in GenAI service research, Emojis, as a form of visual symbols for emotional expression, affect the brain’s cognitive and attentional processing of stimuli. At the same time, based on the model’s perspective that emotions drive cognitive processing pathways, the impact of emojis on cognitive neural activity (N4 component) depends on the nature of the context, while the social function of emotion is modulated by the degree of situational conflict and the depth of information processing.

On the one hand, in high-conflict situations (service failures), emojis activate cognitive analysis and reasoning. When failures occur, people often engage causal thinking mechanisms to avoid repeating mistakes (Dabholkar and Spaid, 2012). At this point, introducing emojis as additional information demands greater cognitive resources and attention allocation from users, often leading them to speculate about the service provider’s intentions. Given the limited cognitive resources available during real-time human-computer interactions, this can induce heightened cognitive conflict.

On the other hand, in low-conflict scenarios (service success), users are more likely to rely on automated emotional processing mechanisms. At this point, GenAI provides users with sufficient, error-free service that aligns with their expectations, avoiding cognitive dissonance. In such cases, emojis serve as supplementary peripheral emotional cues that have minimal impact on users’ cognitive conflicts (Freire et al., 2023).

*H3*: When service fails, the presence of emojis induces greater N4 wave amplitude than their absence.

*H4*: When service succeeds, there is no significant difference in N4 wave amplitude between the presence and absence of emojis.

The P3 component is a positive heading wave mainly distributed in the central to parietal regions of the brain, with a latency of 300–1,000 ms (Polich and Kok, 1995), reflecting the individual’s evaluation and emotional processing of the decision outcome, and is mainly used to assess the user’s emotional attitudes and preferences towards services or products. The EASI model posits that emotional expression signals enhance emotional influence when aligned with the context and audience emotions. This study examines the contextual use of emojis consistent with service outcome information. When GenAI services succeed, users experience positive emotional states. In such scenarios, emojis serve as visual emotional cues. Emojis (e.g., smiley faces) may further stimulate users’ pleasure and identification motivation through a positive emotion superposition effect, with significantly higher P3 wave amplitudes compared to contexts without emojis.

**Figure 2:**
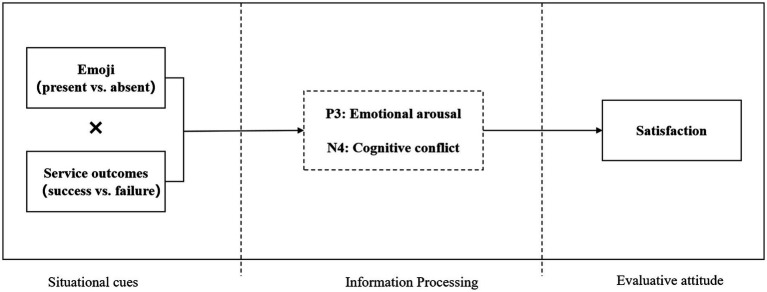
Theoretical model diagram.

Compared to service success, service failure often triggers negative emotional responses from users due to unmet needs. At this point, users are prone to defensive evaluation and heuristic judgments (prioritizing risk signals), leading to heightened vigilance and stronger motivation to resolve issues. The appearance of emojis in such situations can easily make users perceive the interaction as informal and unprofessional, suggesting that GenAI does not prioritize their concerns, thereby provoking more intense emotional reactions. Formally, we propose the following. See Figure 2 for the overall research model.

*H5*: When service fails, the presence of emojis induces greater P3 amplitude than their absence.

*H6*: When service succeeds, the presence of emojis induces greater P3 amplitude than their absence.

## 4 Research methodology

### 4.1 Methods overview

Based on theoretical discussions and hypotheses, we constructed a 2 (Emojis: present vs. absent) × 2 (Service outcomes: success vs. failure) within-subjects experimental design model to examine the impact on user satisfaction. The study employed an ERP experiment using the stimulus-detection paradigm from neuromarketing to test the aforementioned effect mechanisms. Specific methods are detailed in Table 1.

**Table 1:** Method overview.

Core research questions	Hypotheses	Independent variables	Measurement methods /indicators	Dependent variables/observed variables
1. How do emojis and service outcomes influence overall satisfaction?	H1, H2	- Emojis (present vs. absent) - Service Outcome (Success vs. Failure)	Behavioral Measurement: Self-Reported Satisfaction score (key presses)	Satisfaction
2. How do emojis and service outcomes influence users’ cognitive processing?	H3, H4	- Emojis (present vs. absent) - Service Outcome (Success vs. Failure)	ERP Measurement: N4 component amplitude and latency	Cognitive conflict
3. How do emojis and service outcomes influence users’ emotional response processes?	H5, H6	- Emojis (present vs. absent) - Service Outcome (Success vs. Failure)	ERP Measurement: P3 component amplitude and latency	Emotional arousal

### 4.2 Participants

ERP experiments require more than 50 repetitive stimuli per experimental condition for a single subject, so the optimal number of subjects is between 12 and 30 (Wei et al., 2023). In this study, 25 subjects were recruited from the subject pool of the Behavior and Decision Making Laboratory of Huaqiao University to participate in the experiment, all of whom had extensive experience with GenAI. 3 participants with ERP artifacts of more than 25% of the trials were excluded, and the final 22 (10 males, 12 females, age 22.25 ± 2.3 years) university students and graduate students were validly used as subjects. All subjects had normal visual acuity or corrected visual acuity, were right-handed, spoke Chinese as their native language, and had no history of mental illness. All subjects signed an informed consent form before the experiment and were given a certain amount of compensation after the end of the study. The study was approved by the Ethics Committee of Huaqiao University.

### 4.3 Experimental materials

The experimental materials in this study mimic the mainstream GenAI dialog design, and the AI dialog pictures allow participants to better immerse themselves in the scene. The GenAI service response discourse is divided into four categories, service success-with emojis, service success-without emojis, service failure-with emojis, and service failure-without emojis. Examples are as follows: service success-with emojis: I helped you find the drug information you need 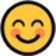; service success-without emojis: I helped you find the drug information you need; service failure-with emojis: the function cannot be explained in detail for you 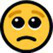; service failure-without emojis: the function cannot be explained in detail for you. There were 25 sentences for each category. This study controls for the consistency between emoji sentiment and service context. In order to exclude the interference of emoji ambiguity, universally recognized emojis with the same meaning as the service outcomes information were adopted, and the interrogation contexts were the main daily application contexts such as healthcare, travel, entertainment, content creation, education and learning, etc., for GenAI applications. All experimental stimulus messages were developed by the authors and evaluated by three experts in the field of human-computer interaction.

### 4.4 Procedure

**Figure 3:**
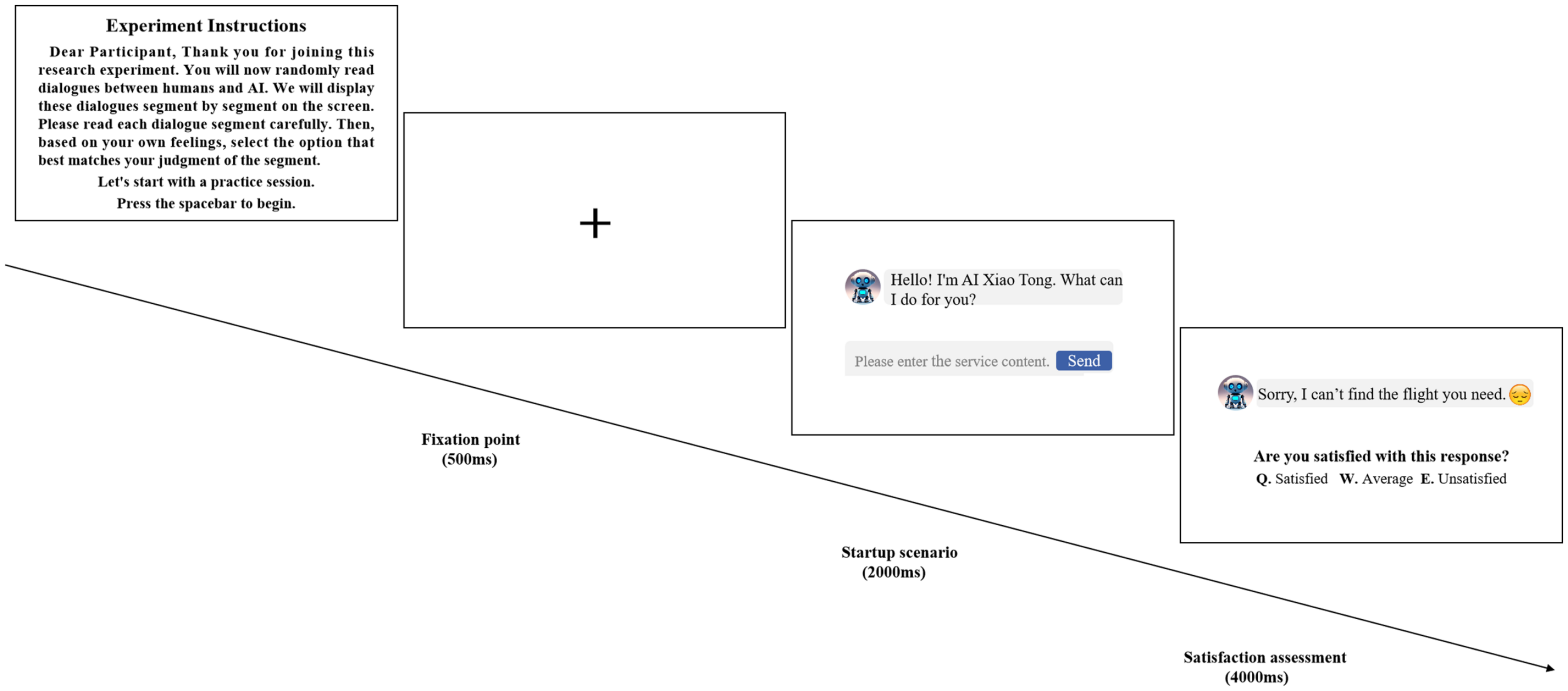
Experimental flow chart.

The stimulus process of the experiment was presented using the E-prime program, and the whole experiment consisted of 300 trials. The experiment was a 2 (service outcome: success vs. failure) × 2 (emoji: present vs. absent) two-factor within-subject ERP experimental method. Experimental participants were comfortably seated in an electrically shielded room with dim lighting and attenuated sound, 100 centimeters away from a computer screen. Participants were guided into the experimental situation by means of a priming paradigm. Each participant had 10 practice sessions to familiarize themselves with the task before the formal experiment, which was divided into 5 groups with a total of 60 trials per group. The flow of the experiment is shown in Figure 3. In a single round of trials, participants were first presented with a 500-ms picture of the gaze point, followed by a 2,000-ms picture of the GenAI conversation, and finally a 4,000-ms presentation of the GenAI service and were asked to judge the level of satisfaction, with the key “Q” being satisfied, the key “W” being average, and the key “E” being unsatisfied.

### 4.5 Data collection and analysis

This experiment used the NeuroScan EEG recording and analyzing system from the United States, and the electrode positions were referred to the 64-lead electrode caps of the international standard 10–20 system expansion, and the vertical electrooculography (VEO) and the horizontal electrooculography (HEO) were recorded at the same time. The impedance at all electrodes was less than 10 kΩ. FCz electrodes were used as on-line reference electrodes, and EEG data were processed through EEGLAB to re-reference the papillae on both sides, with a filtering range of 0.1–40 Hz to exclude the interference of IF, and the effects of artifacts such as concomitant blinks, eye movements, and EMG were excluded through independent component analysis, and the time-domain analysis was selected to be carried out from −200–1,000 ms after the emergence. The N4 component appeared in the central and parietal brain regions (Kutas and Federmeier, 2011), with a time window of 460–490 ms, and six electrodes (C1, Cz, C2, CP1, CPz, CP2) were selected for analysis; the P3 component was located in the frontal-central region and the prefrontal, with a time window of 360–400 ms, and six electrodes (P1, Pz, P2, CP1, CPz, CP2) were selected for analysis.

## 5 Results

### 5.1 Behavioral result

**Figure 4:**
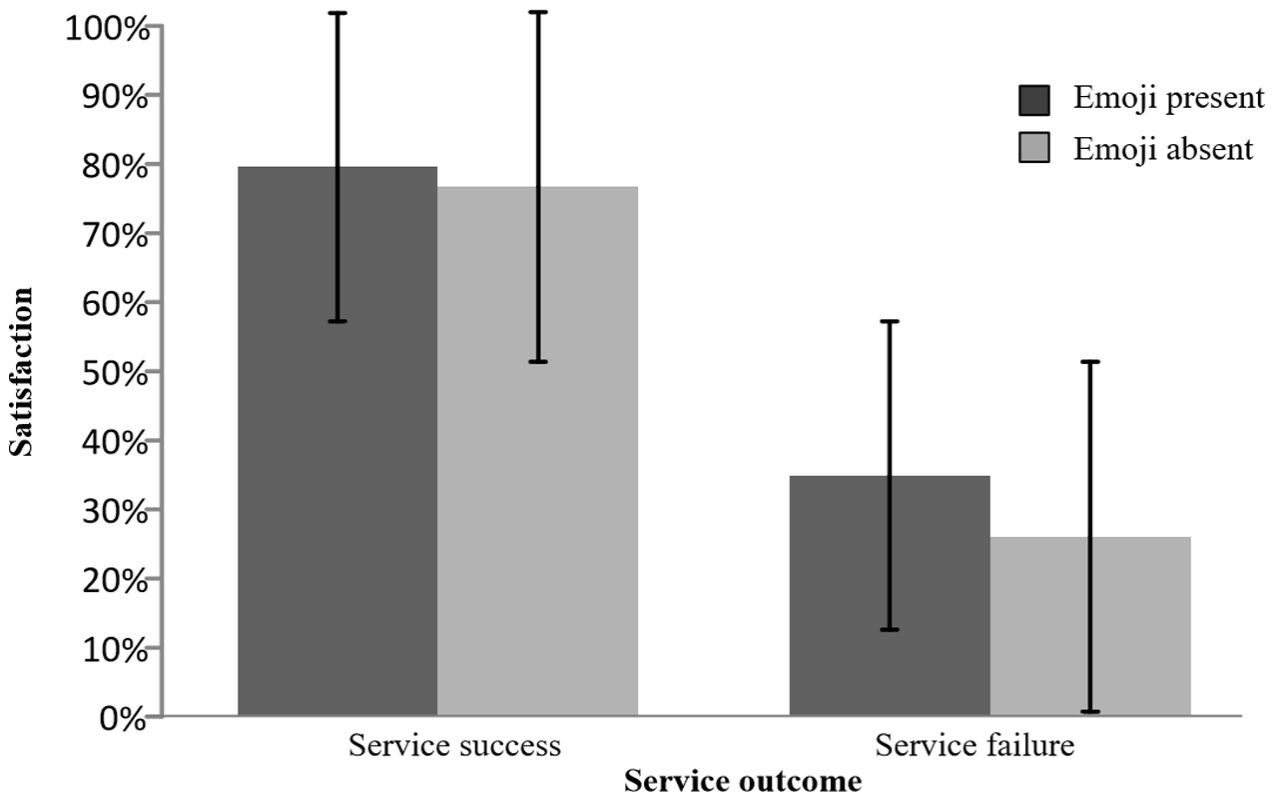
Behavior result graph.

Satisfaction represents the degree to which users recognize the value of GenAI services, measured through their button press responses. A repeated-measures ANOVA revealed a significant main effect of service outcome (*F* (1, 21) = 69.072, *p* < 0.001, ηp^2^ = 0.767), indicating that satisfaction was substantially higher following successful service outcomes (M = 0.782, SE = 0.033) compared to service failures (M = 0.305, SE = 0.042). In contrast, the main effect of emoji was not significant (F (1, 21) = 1.353, *p* = 0.258, ηp^2^ = 0.061). The interaction between emoji and service outcome was not statistically significant (*F* (1, 21) = 3.380, *p* = 0.080, ηp^2^ = 0.139) (see Table 2 and Figure 4). Therefore, H1 and H2 are not supported.

**Table 2:** ANOVA results for behavior result.

Effect	F (1, 21)	*p*-value	ηp^2^	Significance
Emoji type	1.353	0.258	0.061	Not significant
Service outcome	69.072	< 0.001	0.767	Significant
Emoji type × Service outcome	3.380	0.080	0.139	Not significant

### 5.2 ERP results

#### 5.2.1 N4 result

**Figure 5:**
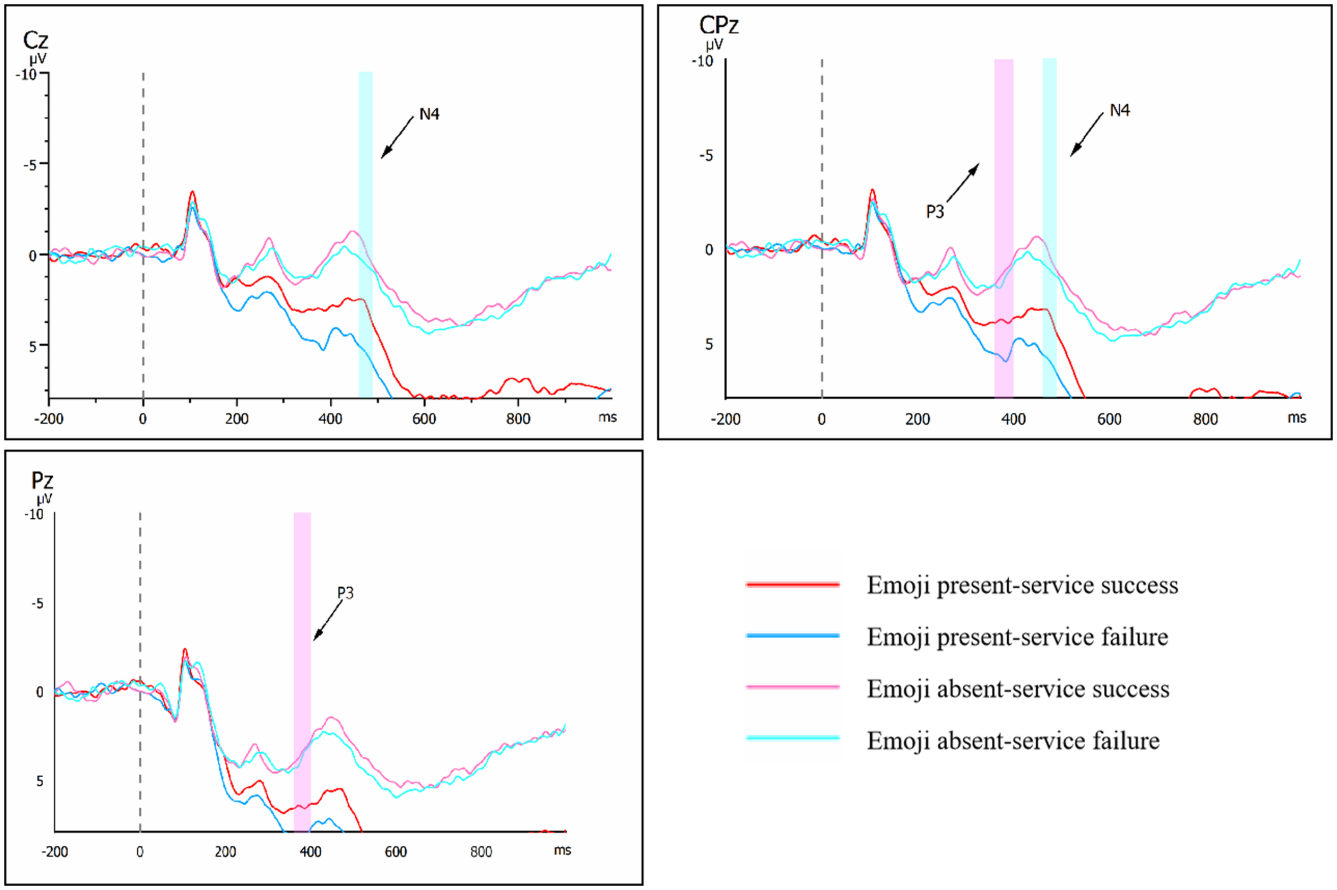
ERP waveform map for each condition.

Within a –200–1,000 ms timeframe, electrodes were used to produce EEG waveforms (see Figure 5). Based on the waveform diagrams, we know that the N4 component time frame is 460–490 ms. Based on existing literature (Naranowicz and Jankowiak, 2025), the N4 was analysed over three central (C1, Cz, C2), and three centro-parietal (CP1, CPz, CP2) electrodes. Furthermore, repeated measures ANOVA was performed on the electrode points of N4 analysis.

As shown in Table 3, for the N4 component, the main effect of emoji was significant (*F* (1, 21) = 33.285, *p* < 0.001, ηp^2^ = 0.613). The main effect of service outcome was also significant (F (1, 21) = 8.020, *p* = 0.010, ηp^2^ = 0.276). The interaction effect between emoji and service outcome was significant (F (1, 21) = 9.348, *p* = 0.006, ηp^2^ = 0.308). Simple effects analysis revealed that under the service success condition, emoji presence had no significant impact on the N4 component (*p* = 0.275). Under the service failure condition, trials with emojis (M = 6.045, SE = 1.194) elicited significantly larger N4 components than those without emojis (M = 1.328, SE = 1.127, *p* = 0.002) (see Table 4). Therefore, H3 and H4 are supported.

**Table 3:** ANOVA results for N4 and P3 components.

Component	Effect	F (1, 21)	*p*-value	ηp^2^	Significance
N4	Emoji type	33.285	< 0.001***	0.613	Significant
	Service outcome	8.020	0.010*	0.276	Significant
	Emoji type × service outcome	9.348	0.006**	0.308	Significant
P3	Emoji type	28.697	<0.001***	0.577	Significant
	Service outcome	7.744	0.011*	0.269	Significant
	Emoji type × service outcome	3.983	0.059	0.159	Marginal

*p < 0.05, **p < 0.01, ***p < 0.001.

**Table 4:** Simple effects analysis for N4 and P3 component.

Component	Service outcome	Emoji type	Mean (μV)	SE (μV)	*p*-value
N4	Service failure	Emoji present	6.045	1.194	0.002**
		Emoji absent	1.328	1.127	
	Service success	Emoji present	3.835	1.255	0.275
		Emoji absent	0.770	1.168	
P3	Service failure	Emoji present	7.443	0.993	< 0.001***
		Emoji absent	3.240	1.058	
	Service success	Emoji present	5.711	1.032	0.001 **
		Emoji absent	2.957	1.018	

*p < 0.05, **p < 0.01, ***p < 0.001.

#### 5.2.2 P3 result

Based on the waveform diagrams (see Figure 5), we know that the P3 component time frame is 360–400 ms. Based on existing literature (Wu et al., 2023), electrodes in the central-parietal (P1, Pz, P2, CP1, CPZ, CP2) regions were selected for P3 analysis, as these areas exhibit high sensitivity to emotional processing. Repeated measures ANOVA was performed on the P3 analysis electrode point.

As shown in Table 3, for the P3 component, the main effect of emoji was significant (F (1, 21) = 28.697, *p* < 0.001, ηp^2^ = 0.577). The main effect of service outcome was also significant (F (1, 21) = 7.744, *p* = 0.011, ηp^2^ = 0.269). The interaction effect between emoji and service outcome was marginal (F (1, 21) = 3.983, *p* = 0.059, ηp^2^ = 0.159). Given the significant interaction effect, we proceeded to conduct an exploratory simple effects analysis. Simple effects analysis revealed that under the service failure outcome, stimuli with emojis (M = 7.443, SE = 0.993) elicited significantly larger P3 amplitudes than those without emojis (M = 3.240, SE = 1.058, *p* < 0.001). Similarly, under the service success outcome, stimuli with emojis (M = 5.711, SE = 1.032) also elicited significantly larger P3 amplitudes than those without emojis (M = 2.957, SE = 1.018, *p* = 0.001) (see Table 4). Therefore, H5 and H6 show a strong trend toward establishment.

## 6 Discussion

### 6.1 Conclusion

The controversy surrounding emoji usage in GenAI services and its impact on user cognition and emotions has become a cutting-edge topic. Based on the EASI model, this study systematically examined how service outcomes and emojis influence user satisfaction and neurocognitive processes from the perspectives of emotional response and cognitive reasoning using ERP technology. The study reached the following conclusions:

(1) Behavioral result: user satisfaction primarily depends on service outcomes, with no significant interactive effect between service outcomes and emojis. Service outcomes (success vs. failure) are the decisive factor for user satisfaction, with satisfaction levels significantly higher in successful scenarios than in failed ones. However, the main effect of emojis and their interaction with service outcomes were not significant. This indicates that in GenAI service interactions, users’ objective assessment of service quality far outweighs their perception of emotive symbols, and emojis fail to effectively moderate satisfaction evaluations directly driven by service outcomes. Therefore, H1 and H2 are not supported.
(2) N4 result: service outcomes and emojis significantly interacted with early cognitive conflict (N4 component). Specifically, in service failure scenarios, emoji presence induced significantly larger N4 amplitudes, requiring users to allocate more cognitive resources for intention attribution and reasoning. In service success scenarios, emojis had no significant effect on N4 amplitude. This result clearly supports H3 and H4.
(3) P3 result: emojis universally amplified emotional stimulus intensity (P3 component), though their interaction with service outcomes reached marginal significance. Exploratory simple effects analysis revealed that stimuli with emojis elicited significantly larger P3 amplitudes than those without, regardless of service success or failure. This indicates emojis, as prominent emotional cues, effectively enhance users’ emotional evaluation processes. Thus, H5 and H6 show a strong tendency toward establishment.

### 6.2 Theoretical contributions

The theoretical value of this study is reflected in the following aspects: firstly, this study extends research on the EASI model in the field of GenAI services, providing a crucial explanatory framework for understanding the boundaries of emotional expression in human-computer interaction. Consistent with existing findings, this study reveals that GenAI’s use of emojis enhances users’ cognitive and emotional responses. Furthermore, in real-time human-computer interactions, the impact of emojis may be more pronounced in high-conflict scenarios such as service failures, while exhibiting weaker or even negligible effects in low-conflict situations like successful service delivery.

Second, this study employs high-temporal-resolution ERP technology to capture users’ cognitive processing and immediate responses in real time during interactions with GenAI. This methodology provides more objective and precise evidence and methodological insights for understanding and evaluating users’ genuine attitudes and reactions during GenAI interactions.

Finally, the study reveals a decoupling between behavioral and neural levels in real-time GenAI human-computer interactions. Findings indicate that emojis trigger early cognitive conflict and amplify emotional responses, yet these implicit processes do not necessarily translate into shifts in user attitudes or evaluations. This “behavior-neural” decoupling demonstrates that users’ implicit processing of emotional cues in real human-computer interactions is complex and multi-stage. This provides insights for understanding the phenomenon of users’ “discrepancy between words and actions” in human-computer interaction.

### 6.3 Practical implications

This study also makes some practical suggestions for the design and optimization of GenAI services. First, in GenAI service management, enterprises need to follow the principles of prioritizing the quality of functions and results. Developers and service providers should prioritize the optimization of core service success (e.g., improving algorithmic accuracy, enhancing algorithmic self-learning and reviewing ability) and reducing service failures. When services fail, minimize the use of emojis that may trigger cognitive dissonance. When services succeed and brand personification is required, tie emojis to interpretable cues to avoid emotional dissonance, where neural responses are prominent but behavioral responses are muted. This approach reduces unnecessary design costs and prevents users from “voting with their feet.” Second, this study also expands new perspectives and tools for users to evaluate GenAI services, including satisfaction indicators, EEG activity monitoring, etc., which provides a scientific basis for service providers to optimize and improve and adjust their strategies. Service providers can fully integrate research findings and technologies such as ERP and eye-tracking to precisely and scientifically optimize service outcomes and deliver emotional value. Finally, service providers and researchers need to strengthen the ethical boundaries and contextual sensitivities of symbol use (e.g., prioritized message clarity in the medical, financial, and educational domains) in GenAI training.

### 6.4 Limitations and prospects

Some limitations must be acknowledged, and follow-up studies are required. First, the sample size was relatively small and primarily comprised college students, resulting in limited statistical power and external validity. Although college students represent a major user group of GenAI and exhibit high representativeness, and the statistical power of the 22 samples in this study exceeded 0.80 based on post-hoc power analysis using G*Power 3.1 (*f* = 0.30, *α* = 0.05), the homogeneity of the sample in terms of age and cultural background future research should expand sample size and diversity (e.g., cross-cultural differences between Chinese and foreign groups, diverse professions) to enhance the external validity of findings. Second, this study examined only the most common combination of “text information + static emojis.,” As GenAI technology rapidly evolves and user needs diversify, future research should explore the influence mechanisms of more complex emoji characteristics (e.g., static/dynamic, position, sequence) and service outcomes (e.g., process results, final results) on user experience and evaluations across different contexts (e.g., emotional comfort, information consultation). Finally, constrained by ERP methodology limitations, this study focused solely on users’ immediate neural and behavioral responses during GenAI interactions without tracking long-term effects. Future research aims to develop a multi-round interaction tracking framework, collecting behavioral logs, trust scales, and reuse decisions simultaneously at baseline, 1 week, and 1 month post-interaction. Latent variable growth models will be employed to characterize the dynamic trajectories of emojis and service outcomes on users’ long-term engagement.

## Data Availability

The data presented in the study are deposited in the Open Science Framework (OSF) repository, accession number “DOI 10.17605/OSF.IO/KU39Z”.
